# Surgical Management of Atraumatic Rupture of Splenic Artery Aneurysm with Spleen Preservation in a Regional Australian Hospital

**DOI:** 10.1155/2023/5738806

**Published:** 2023-03-06

**Authors:** Emma Jane Hamilton, Samuel Ngugi, Rasika Kotakadeniya

**Affiliations:** ^1^Department of General Surgery, Bundaberg Base Hospital, Bundaberg, QLD, Australia; ^2^University of Queensland, St Lucia, QLD, Australia

## Abstract

A 41-year-old male presented to the emergency department of a regional Australian hospital with chest and abdominal pain. He became rapidly haemodynamically unstable and was diagnosed with a ruptured splenic artery aneurysm and large volume hemoperitoneum. Due to the regional location of our small hospital, endovascular services are not available and the patient required emergency laparotomy. At laparotomy, a 2 L hemoperitoneum was evacuated, and the bleeding splenic artery aneurysm was identified and controlled. The aneurysm was approached with a unique technique via division of the gastro colic omentum to enter the lesser sac. This allowed adequate exposure of the splenic artery and proximal and distal control of the vessel was achieved. Adequate perfusion to the spleen was preserved by this surgical technique and splenectomy was therefore not required. This study details the management of this patient, details of the interoperative technique, and a discussion regarding splenic artery aneurysms. Splenic artery control and ligation without splenectomy may be considered in appropriate patients and splenectomy is therefore not always required in cases of hemodynamic instability where open surgical management is performed.

## 1. Case Report

A 41-year-old male presented to the emergency department of a regional Australian hospital with sudden onset severe chest and abdominal pain. On arrival, the patient was haemodynamically stable, but became rapidly unresponsive with oxygen saturations of 85%, tachycardia to 140 beats per minute, unrecordable blood pressure, and a Glasgow Coma Scale of 8 (eyes 3 points, voice 1 point, and motor 4 points). Bedside, ultrasound scan yielded intra-abdominal free fluid in the left upper quadrant. The patient was resuscitated sufficiently to allow prompt computerized tomography (CT) angiogram. He had no history of trauma, falls, pancreatitis, or known hypertensive crisis, and no knowledge of any previous aneurysms.

**Figure 1:**
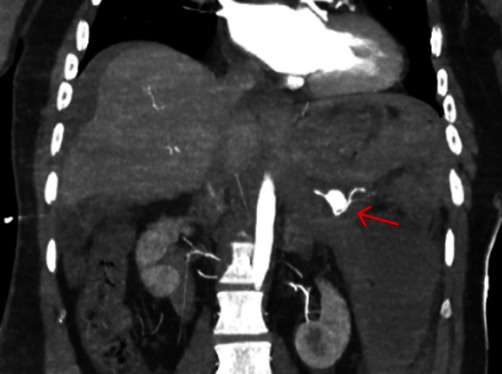
Large volume hemoperitoneum with active contrast extravasation from splenic artery on abdominal CT angiogram.

**Figure 2:**
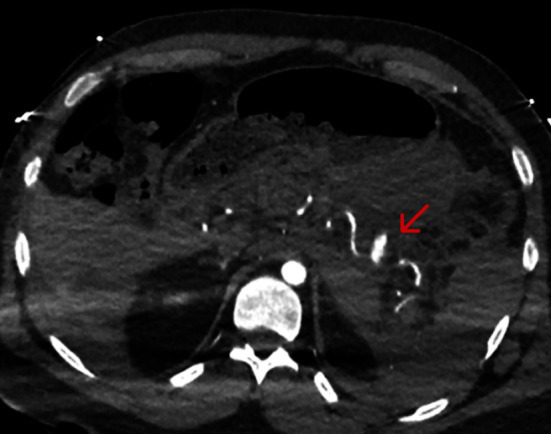
Splenic artery aneurysm with active contrast blush on abdominal CT angiogram.

CT angiogram demonstrated a large left upper quadrant intraperitoneal and retroperitoneal haematoma with dense peritoneal free fluid consistent with hemoperitoneum (Figure 1). A splenic artery aneurysm at least 28 mm × 16 mm was seen with active contrast extravasation (Figure 2).

In our regional centre, there are no interventional radiology or endovascular services available; hence, the patient underwent emergency laparotomy where a 2 L hemoperitoneum was evacuated. The bleeding splenic artery aneurysm was approached via division of the gastro colic omentum and reflection of the stomach superiorly to allow exposure of the lesser sac. This allowed adequate visualisation of the length of the splenic artery and the ruptured aneurysm mid-way along the artery was easily seen. Following this exposure, control of the splenic artery vessel at two points was achieved via suture ligation: the splenic artery proximal to the aneurysmal sac and distal to the aneurysmal sac were both secured, resulting in ligation of the artery at these two points. This was sufficient to achieve haemostasis. Due to the exposure technique via division of the gastro colic omentum, the short gastric and left gastro epiploic arteries were undisturbed and left intact allowing collateral circulation to the spleen to persist despite ligation of the splenic artery. Perfusion to the spleen appeared preserved and splenectomy was not required to be performed.

**Figure 3:**
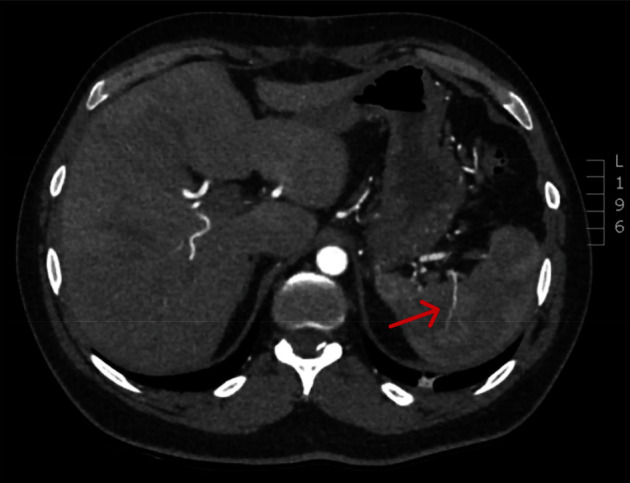
CT angiogram confirming adequate collateral perfusion of the spleen 3 weeks post-laparotomy.

The patient was admitted to the intensive care unit postoperatively for monitoring and made an unremarkable recovery. A surgical drain placed into the left upper quadrant of the abdomen at the time of operation had minimal output with no evidence of ongoing bleeding and was able to be removed day 2 postoperatively. On post-operative day 3, the patient remained very well and was fit for discharge home after an ultrasound scan confirmed continuing splenic perfusion and organ viability, though noted some surrounding residual haematoma. Three weeks postoperatively, the patient remained well and underwent surveillance CT angiogram, which confirmed ongoing sufficient collateral perfusion of the spleen and viability of the organ, with a resolving peri-splenic haematoma also seen (Figure 3). The differential diagnosis for the cause of the peri-splenic haematoma includes inadequate evacuation of haematoma at time of operation, iatrogenic damage to collateral vessels resulting in minor bleeding postoperatively, or inadequate control of the splenic artery. The post-operative drain had minimal output and the haematoma was reduced in size on repeat imaging; therefore, inadequate evacuation of an already formed clot from the time of operation is the most likely cause. At review 6 months post-procedure, the patient remained well with no latent complications.

## 2. Discussion

Visceral artery aneurysms can be either true aneurysms or pseudoaneurysms that affect any branch of the abdominal aorta defined as an abnormal dilation of the artery 50% greater than the baseline or normal vessel diameter [1]. 60% of visceral artery aneurysms arise from the splenic artery, 20% from the hepatic artery, 5–8% from the superior mesenteric artery, 4% from the coeliac artery, 2–4% gastric or gastroepiploic, and 2–3% jejunal, ileal, and colic artery aneurysms [2]. In addition, whilst splenic artery aneurysms are the most common site of visceral artery aneurysm it overall remains a rare condition with incidence in the general population believed to be around 0.8% [3].

Splenic artery aneurysms have an annual rate of rupture between 2% and 10% [1, 4] in asymptomatic patients increasing to 76–83% in symptomatic patients [1]. Mortality from spontaneous rupture of a splenic artery aneurysm in non-pregnant patients ranges from 25% to 40% with higher mortality rates in pregnant women [1]. The aetiological cause is not able to be established in all cases, but contributing factors include atherosclerosis, multiple pregnancies, portal hypertension, acute or chronic pancreatitis, vasculitis, and congenital anomalies including fibromuscular dysplasia or collagen vascular diseases [1, 4, 5]. A literature review of all documented splenic artery aneurysm cases at the time by Tessier et al. found that chronic pancreatitis was the presumed cause in 46% of patients, trauma the cause in 29% of patients, and the next most common cause was unknown or no reported cause occurring in 14% of the documented cases [6]. Splenic artery aneurysms can occur as true aneurysms, whereby the arterial wall is weakened by a number of potential causes including atherosclerosis (32%), medial degeneration or dysplasia (24%), abdominal trauma (10%), hypertension, connective tissue disease, or necrotizing vasculitis [1]. Pseudoaneurysms of the splenic artery have a different aetiology, whereby there is rupture of vessel intima and media resulting in a periarterial haematoma contained by the adventitia surrounding the vessel. These are more commonly caused by iatrogenic trauma or inflammatory processes, such as chronic pancreatitis [1].

Emergency management of rupture can be via endovascular vessel embolization or splenectomy via emergency laparotomy. A study by Martinelli et al. published in 2019 recommended that endovascular approach should be considered first line even in emergency settings [7]; however, in our small Australian regional town endovascular management is not available with the nearest capable centre being 295 km away. The patient was deemed too unstable to await interhospital transfer; therefore, surgical management was the only readily available appropriate option in this case.

The spleen is a well perfused organ with extensive collateral circulation via the superior mesenteric, pancreatic, and left gastric arteries as well as via the short gastric arteries. An anterior approach to splenectomy will require ligation of the left gastro-epiploic and short gastric arteries, which will eliminate most of the collateral circulation once the splenic artery is controlled; therefore, necessitating splenectomy [8]. These patients who undergo splenectomy will have modified immunological function and require long term antibiotics, vaccinations, and are at increased risk of overwhelming post-splenectomy infections. There is no available literature detailing potential long-term complications post-spleen preserving open splenic artery ligation. There is however many cases detailing endovascular ligation of splenic artery aneurysms (angioembolization), which retains the spleen in situ. Complications associated with this procedure include infarction of the spleen, splenic abscess formation, pseudocyst formation, vascular injury, pancreatitis, or intestinal perforation [9]. These complications that are secondary to splenic hypoperfusion, due to alteration in blood supply are theoretically possible in the described case though no such complications had arisen by 6 months post-procedure.

In this case, the splenic artery was approached in the lesser sac at the upper border of the pancreas by dividing the gastro colic omentum. Direct ligation of the splenic artery allowed haemorrhage control whilst preserving collateral perfusing vessels. The spleen was thus able to remain situ, which is a unique approach in a haemodynamically unstable patient. Splenic artery control and ligation without splenectomy may be considered in appropriate patients and splenectomy is therefore not always required in cases of haemodynamic instability.

## Data Availability

Data supporting this research article are available from the corresponding author or first author on reasonable request.
